# Variations of Flavone Glycosides Profile Underscore the Necessity of Quality Control of Prepared Microctis Folium Slices

**DOI:** 10.1155/2020/8887489

**Published:** 2020-11-26

**Authors:** Kun-Ping Li, Min Yuan, Chang-Le Liu, Ze-yu Liang, Tian-Ling Pan, Jiao Guo

**Affiliations:** ^1^Institute of Chinese Medicinal Sciences, Guangdong Pharmaceutical University, Guangzhou 510006, China; ^2^Guangdong TCM Key Laboratory for Metabolic Diseases, Guangzhou 510006, China; ^3^Second People's Hospital of Huangdao District, Qingdao 266000, China

## Abstract

Microctis Folium (MF), the dried leaves of *Microcos paniculata*, is widely used as a medical and food dual-purpose herb in South-east Asia and China. However, the quality control of MF is not well studied. A simple and reliable quality control method was urgently needed for its growing usage. Herein, at first, its main active components were identified by UPLC-ESI-qTOF-MS, and a representative MF flavone glycosides profile consisting of ten compounds was illustrated, which is the most detailed one up to now. Successively, using vitexin as the reference substance, a novel QAMS method with HPLC for quantification of the ten identified flavone glycosides was developed and methodologically validated. Furthermore, making use of the abovementioned QAMS method, quantitative profiling of 21 batches of prepared MF slices collected from different hospital pharmacies were performed. As a result, the total contents of ten flavone glucosides and the content of specific compound showed obvious variations. Using the ten compounds' contents dataset, the 21 batches of samples were divided into two distinct clusters by HCA. In sum, our results indicated that it was of great importance to take quality control of prepared MF slices and we presented a robust and simple method for their quantitative determination, which should be beneficial for the quality control of MF and its derived products.

## 1. Introduction

*M. paniculata*, also known as *Grewia nervosa*, is a medicinal and edible plant widely distributed in South China, Indian, Indonesia, Myanmar, and so on [1]. It has been reported that the constituents of the roots, stems, barks, and fruits of *M. paniculata* possess *α*-glucosidase inhibitory, free-radical scavenging, antinociceptive, antidiarrheal, anti-inflammatory, analgesic, and antipyretic activities [2–5]. Microctis Folium (MF), the dried leaves of *M. paniculata*, has been added to Chinese Pharmacopeia (Ch.P.) as a kind of traditional herbal medicine. MF is described to have positive effects on fever, jaundice, heat-stroke, indigestion, and diarrhea [6]. It is generally used for producing some Chinese patent drugs and some kinds of Chinese herbal tea beverage which are as popular as cola in Western countries. In the past few years, some researches have proved the hepatoprotective, antipyretic, and analgesic effect of MF [7–10]. Phytochemical studies show that MF has triterpenoids, alkaloids [11], and, by virtue of its high content of flavonoids, ethanol extract of MF possessing excellent antioxidant and anti-inflammation activities [12–14]. Hence, making use of the flavonoids fraction of MF as functional dietary supplements has attracted much attention. However, knowledge on its active components qualitative and quantitative analysis is lacking, and the quality control of prepared MF slices is not well investigated, which limits its further development and utilization as functional foods.

Nowadays, liquid chromatography coupled with quadrupole orbital ion trap mass spectrometry (LC/Q-Orbitrap-MS) and liquid chromatography coupled with electrospray ionization tandem time of flight mass spectrometry (LC-ESI-qTOF-MS) are two convenient ways to qualitatively analyze the components in some herbal or botanical medicine [15–17]. Although liquid chromatography coupled with electrospray ionization triple quadrupole mass spectrometry (LC-TQ-MS or LC-QQQ-MS) are powerful method for quantitatively profiling of biosamples [18], it means high cost and advanced technological assistance. Hu et al. [19] qualitatively deduced thirty-two compounds in MF including ten flavans, eight flavone O-glycosides, eight flavone C-glycosides, five lignan glycosides, and one megastigmadien glycoside by HPLC-DAD-ESI-MS and quantified three of the identified flavone glycosides. As our previous research on the protective effects of MF flavonoids fraction against acute lung injury was induced by LPS, eight apigenin C-glycosides such as vicenin-2, isoshaftoside, shaftoside, vitexin, vicenin-1, isovitexin, violanthin, and isoviolanthin were identified by UPLC-ESI-qTOF-MS [14]. Although LC-MS is powerful for qualitative identification of natural products, high performance liquid chromatography (HPLC) is the most simple but valid method used for determination of the content of most of the plant secondary metabolites, including flavonoids. Closely following Li et al. [20] who analyzed the HPLC fingerprint of MF flavonoids, Chen et al. [21] quantified five components including ferulic acid, vitexin, isovitexin, kaempferol-3-O-*β*-D-rutinoside and narcissoside by HPLC, and Chen et al. [6] also determined three flavone glycosides in MF, namely, vitexin, isovitexin, and isorhamnetin 3-O-*β*-D-rutinoside by HPLC. All the aforementioned literatures proved that HPLC was suitable for quantifying the flavonoids in MF, but one more convenient method covering more active components is still desired.

When HPLC is used to simultaneously determine multicompounds in herbal or botanical products, two typical methods are usually taken into account. The first method, also the most common one, is called external standard method (ESM), which needs every reference substance for the analysis of each objective component. This method is the most acceptable one by most researchers. Moreover, it has been acknowledged by both United States Pharmacopeia (USP) and Ch.P. for quality control of herbal drugs. The other method requires only one single reference substance to determine multicomponents, called quantitative analysis of multicomponents by single marker (QAMS). Comparing with ESM, QAMS reduces the expenditure on reference substances and shortens the operation time, especially suitable for quantification of a serial of analogues [22]. QAMS method has been used for the quality control of some herbal drugs, such as *Panax notoginseng* [23], *Andrographis herba* [24], and *Radix astragali* [25].

In the present study, in order to delineate a representative flavone glycosides profile, UPLC-ESI-qTOF-MS was used firstly to identify the main flavone glycosides in MF according to the retention time (*t*_*R*_), mass-to-charge ratio (*m*/*z*), and fragmentation patterns. Then, using vitexin as the reference substance, a novel QAMS method with HPLC for quantification of the identified flavone glycosides was developed and methodologically validated. Successively, by making use of the abovementioned QAMS method, quantitative profiling of 21 batches of prepared MF slices collected from different hospital pharmacies were carried out. All our efforts aim to provide some evidence and suggestions for establishing a complete quality control system for MF.

## 2. Experimental

### 2.1. Reagents and Chemicals

Vicenin-2, schaftoside, vitexin, 2”-O-rhamnosylvitexin, isovitexin, astragalin, narcissoside, and nicotiflorin (used as reference substance) were purchased from Chemface Co. Ltd. (Wuhan, China). Isoschaftoside and isoviolanthin were isolated in our lab from the ethanol extract of MF, and the purity of which was determined by analytical HPLC, and the chemical structure was confirmed by HRMS, ^1^H-NMR, and ^13^C-NMR (Figures S1 and S2).

Methanol (HPLC grade) was purchased from Merck Chemicals (Darmstadt, Germany). Formic acid (95%) and phosphoric acid were from Sigma-Aldrich (St. Louis, MO, USA). Deionized water was obtained by a MilliQ apparatus (Millipore, Milford, MA, USA).

### 2.2. Preparation of Reference Substance Solution

The stock solution of vicenin-2 (0.625 mg/mL), isoschaftoside (0.750 mg/mL), schaftoside (0.656 mg/mL), vitexin (0.731 mg/mL), 2”-O-rhamnosylvitexin (0.930 mg/mL), isovitexin (0.738 mg/mL), isoviolanthin (1.329 mg/mL), nicotiflorin (0.731 mg/mL), astragalin (0.706 mg/mL), and narcissoside (0.806 mg/mL) was prepared by accurately weighing appropriate amount of abovementioned reference substance in a 25 mL volumetric flask and dissolved it with methanol, respectively. From the stock solutions, working mixed reference substance solution was prepared by mixing and diluting with methanol appropriately. The working solution was filtered through 0.45 *μ*m membrane filter.

### 2.3. Preparation of Sample Solution

The prepared MF slices were collected from different hospital pharmacy during summer 2016. All the samples were dried at 60°C for 24 h in drying oven (Liedong electronic Co. Ltd., Foshan, China) and pulverized to fine powder prior to analysis. Sample solutions were prepared as previously described [20]. Briefly, for each sample, 2.5 g of the powdered MF was extracted with MeOH : water (70 : 30) for 1 h with the sonication at room temperature. The final volume was fixed to 50 mL. The extract was filtered by 0.45 *μ*m membrane filter prior to HPLC analysis. At the same time, 100 *μ*L of each filtrate was pooled together as a mixed sample subjected to qualitative analysis by UPLC-ESI-qTOF-MS and HPLC methodological validation.

### 2.4. UPLC-ESI-QTOF-MS Analysis

The qualitative identification was carried out on 1290 UPLC-6545-qTOF-MS with Masshunter workstation B.07.00 (Agilent Corporation, CA, USA). Chromatographic separation was achieved on an Agilent Zorbax Eclipse plus C18 column (2.1 × 100 mm, 1.8 *μ*m particle size). The mobile phase was composed of solvent A (methanol) and solvent B (0.05% formic acid-water) with a gradient elution (0–7.6 min, 85 ⟶ 60% A; 7.6–10 min, 60 ⟶ 60% A; 10–12 min, 60 ⟶ 30% A; 12–15 min, 30 ⟶ 20% A; 15–16 min, 20 ⟶ 0% A; 16–20 min, 0 ⟶ 0% A). The flow rate of mobile phase, the injection volume, and the column temperature were 0.2 mL/min, 2 *μ*L, and 35°C, respectively.

The MS analysis was performed under the following operation parameters: nitrogen was used as both drying gas (8 L/min, 300°C) and sheath gases (11 L/min, 350°C), the capillary voltage was 3500 V, the nozzle voltage was 1000 V, and the nebulizer pressure was set at 35 psi. Data acquisition from *m*/*z* 100 to 1200 under the extended dynamic range (2 GHz) scan mode, with a scan speed of 3 scans/second. The collision energy was adjusted from 15 to 35 V automatically.

### 2.5. HPLC Conditions

HPLC analysis was performed on a Shimadzu LC-10AVP HPLC system consisted with two pumps, a SIL-20A prominence auto-sampler and an SPD-10AVP detector coupled with Shimadzu Labsolutions (Shimadzu, Japan). The conditions were set as previously described [14] with slight modification. Briefly, chromatographic separation was carried out on a Kromasil C18 column (250 mm × 4.6 mm, 5 *μ*m particle size, AkzoNobel, Sweden). Mobile phase consisted of solvent A (methanol) and solvent B (0.2% phosphoric acid). Gradient elution was programmed as follows: 0–5 min, 82% ⟶ 70% (B); 5–45 min, 70% ⟶ 55% (B); 45–55 min, 55% ⟶ 80% (B). The UV absorption was monitored at 276 nm. The column temperature was maintained at 35°C. The flow rate was set at 1.0 mL/min and the sample injection volume was 10 *μ*L. Peaks were assigned according to their retention time and also by coelution with reference substances under the same chromatographic conditions.

### 2.6. HPLC Method Validation and QAMS Suitability

To verify the rationality of the HPLC method, we conducted a series of validation tests, which assessed the system's suitability, linearity, system precision, accuracy, and stability. Both ESM and QAMS were used to calculate the amount of the identified compounds in each sample. The suitability of QAMS was assessed by comparing the quantitative results calculated by ESM and QAMS.

#### 2.6.1. HPLC System Suitability

10 *μ*L of the selected sample solution and mixed reference substance solution were injected into the HPLC system, respectively. The system suitability was assessed by the separation of the peaks in the chromatography.

#### 2.6.2. Linearity

The calibration curves were constructed with five different concentrations. The linear regression equation was drawn by taking the sample injection amount as the abscissa and the peak area as the ordinate. The peak area of the reference substances was mainly considered for plotting the linearity graph. The linearity was evaluated by linear regression analysis, which was calculated by the least square regression method.

#### 2.6.3. System Precision, Stability, and Repeatability

System precision was assessed by repeated injections (*n* = 6) of the mixed reference substances solution. The relative standard deviation values (RSD, %) of peak area as well as the similarity of chromatogram were determined. The acceptance criterion was ±2% for the RSD.

The stability of the solution was determined by using the MF sample for short-term stability by keeping at room temperature for 24 h and then analyzing. Representative samples were injected at 0, 4, 8, 12, and 24 h for analysis, respectively. The stability of the instrument detection was judged by the chromatogram similarity at different time points and the RSD of the peak area.

To confirm the repeatability, six different working solutions prepared from the same prepared MF slices (S3 was randomly selected) were analyzed in parallel. The repeatability of manual operation was the RSD of each peak area. The acceptance criterion was ±2% for the RSD.

#### 2.6.4. Calculation of Relative Conversion Factor

Vitexin was selected as the reference compound because it was abundant in each sample. Moreover, it is stable, is easily obtainable, and can be well separated under the conventional chromatographic conditions. Using vitexin as a reference substance, *f*_*x*_ was calculated by the ratio of the peak area and the concentration between the analyte and vitexin (see (1)) [23–25]. Based on the specific *f*_*x*_ of objective compound, its concentration (*C*_*x*_) in the sample solution could be calculated according to the following formula (see (2)).

(1) $$\begin{array}{c} f_{x}=\frac{A_{s}/C_{s}}{A_{x}/C_{x}}, \end{array}$$

(2) $$\begin{array}{c} C_{x}=\frac{A_{x}\times C_{s}}{A_{s}}\times f_{x}. \end{array}$$

Among these equations, *f*_*x*_ is the average relative conversion factor of determined compound in MF. *A*_*s*_ is the peak area of reference substance and *C*_*s*_ is the concentration of reference substance. *A*_*x*_ is the peak area of determined component in the standard solution or in sample solutions, and *C*_*x*_ is the concentration of determined compound in the single standard solution or in sample solutions.

#### 2.6.5. QAMS Suitability

The quantitative results by ESM of ten identified compounds in 21 batches of samples, namely, vicenin-2, schaftoside, vitexin, 2”-O-rhamnosylvitexin, isovitexin, astragalin, narcissoside, and nicotiflorin, were calculated according to the standard curves constructed in the above process. At the same time, the content of the abovementioned ten flavone glycosides except for vitexin were also quantified by QAMS method described above. Then the suitability of QAMS method was evaluated by comparing the data obtained by both of the two methods. Relative error (*δ*) < 5% was considered that the two methods had the same efficiency [26].

### 2.7. Statistical Analysis

All the values were represented as means ± standard deviation (SD). Student's *t*-test was carried out and *p* < 0.05 was considered to be statistically significant. SPSS Statistics 21.0 software (IBM, IL, USA) and Graphpad Prism 6.0 software (GraphPad, CA, USA) were used for graphics.

## 3. Results and Discussion

### 3.1. A Representative MF Flavone Glycosides Profile Was Delineated

The components in TCM can be identified according to *t*_*R*_, accurate *m*/*z,* and characterized fragmentation pattern with reference substances by UPLC-ESI-qTOF-MS/MS [27]. In this study, ten flavone glycosides were well separated within 20 min by using the aforementioned chromatographic conditions (Figure S3). All of the peaks showed obvious parent ions [M-H]^−^ in the negative ion mode. Using all the information we collected, a representative flavone glycosides profile in MF was well illustrated.

**Figure 1:**
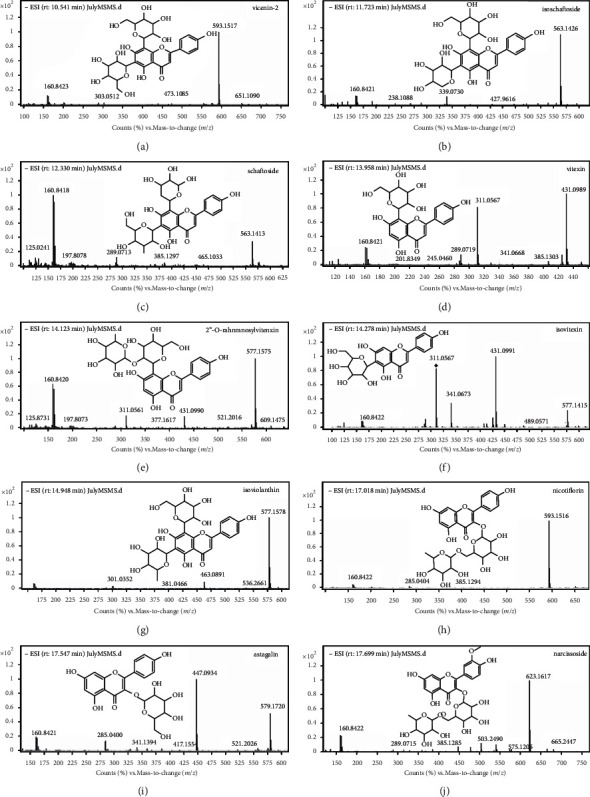
Chemical structures and mass spectra of ten flavone glycosides in Microctis Folium. The ten compounds were vicenin-2, isoschaftoside, schaftoside, vitexin, 2”-O-rhamnosylvitexin, isovitexin, isoviolanthin, nicotiflorin, astragalin, and narcissoside.

Generally, flavonoids consisted of a chromane-type skeleton, forming a C_6_-C_3_-C_6_ skeletal structure. They often possess hydroxyl in positions 3, 5, 7, 3', 4', and 5' [28]. As discovered in many other plants, the flavone glycosides in MF were presented as C- or O-glycosides (Figure 1). The ten flavone glycosides were all hydroxylated in positions 5 and 7. Furthermore, the C-glycosides have sugar substituent directly bound to 6-C and 8-C, whereas the O-glycosides have sugar substituents located at position C-3 [29, 30]. Different structures always lead to various fragmentation pathways. Except for the fact that cleavage of the sugars bonded to the rings is the easiest fragmentation pathway, Rijike et al. [31] made a point that the major fragmentation pathways of C-ring are the cleavage of C-O and C-C bonds at the positions 1/3, 0/2, 0/3, 0/4, or 2/4. As for flavonoids with two hydroxyls located at positions 5 and 7, the cleavage of 2/4 was regarded as the most common way [32].

**Figure 2:**
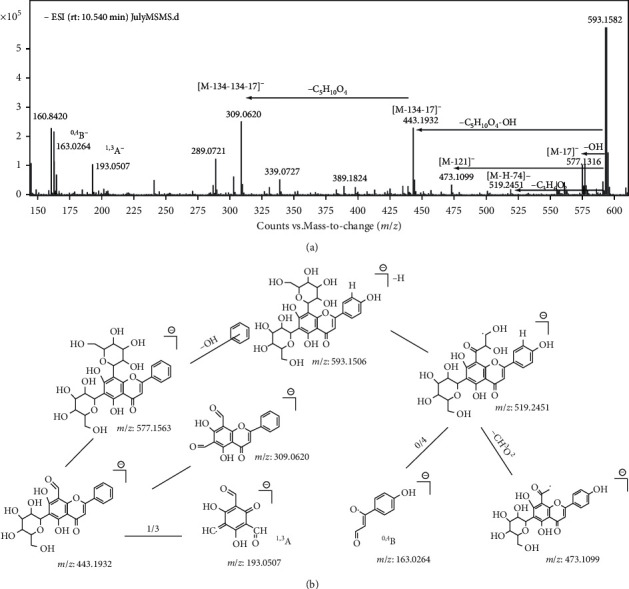
ESI (-)-MS/MS product-ion spectrum (a) and fragmentation pattern (b) of vicenin-2 (*m*/*z* 593.1532).

**Figure 3:**
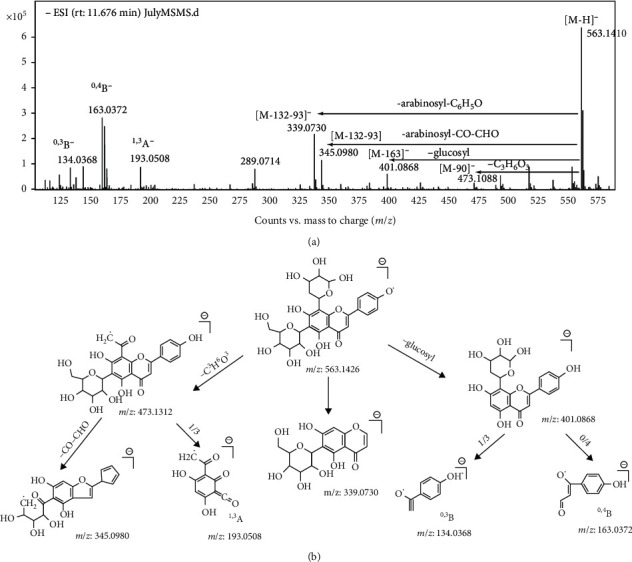
ESI (-)-MS/MS product-ion spectrum (a) and fragmentation pattern (b) of isoschaftoside (*m*/z 563.1410).

To identify these compounds, the experiences raised in previous researches provided an enlightening guide. The fragmentation patterns of flavone glycosides were similar to each other. The potential fragmentation pathways of vicenin-2 and isoschaftoside were shown sophisticatedly in Figures 2 and 3. According to the information that UPLC-ESI-qTOF-MS profiling presented in Figure 1, ten flavone glycosides were identified unequivocally (Table 1). Compared with the previous researches [14, 19], the quantitative flavone glycosides profile of MF presented by us was the most comprehensive one up to now. It could be a foundation for the future study on the utilization of the flavone glycosides fraction of MF.

### 3.2. A Novel QAMS Method for Quantitative Profiling of Ten Flavone Glycosides in MF Was Developed

#### 3.2.1. System Suitability and Linearity

**Figure 4:**
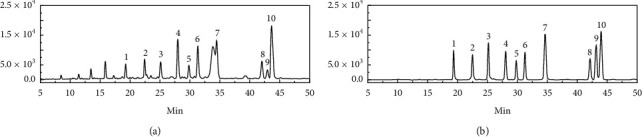
Representative HPLC chromatograph of Microctis Folium (a) and mixed reference substances solution (b): vicenin-2 (1), isoschaftoside (2), schaftoside (3), vitexin (4), 2”-O-rhamnosylvitexin (5), isovitexin (6), isoviolanthin (7), nicotiflorin (8), astragalin (9), and narcissoside (10).

As shown in Figure 4, ten flavone glycosides in MF and mixed reference substances solution can be separated well, indicating that the suitability of the system met our testing requirements.

The calibration curves of vicenin-2, isoschaftoside, schaftoside, vitexin, 2”-O-rahmnosylvitexin, isovitexin, isoviolanthin, nicotiflorin, astragalin, and narcissoside were listed in Table 2. All *r* values obtained using linear regression were about 0.999, which show that the 10 compounds presented good linear at relevant ranges, respectively.

#### 3.2.2. Precision, Repeatability, and Stability of the HPLC Analytical Method

RSD of relative integral area of each peak of mixed ten reference substances was calculated. The results showed that the values were all less than 2.0%, indicating that the instruments had good precision. RSD values of peak area of ten compounds in the randomly selected samples injected at 0, 3, 6, 12, 18, and 24 h were all less than 2%. The similarity of HPLC fingerprint profiles acquired at above six different time spots was above 0.98, which demonstrated a good stability in the tested MF extract within 24 h. RSD values of peak area of 10 compounds in six different working solutions prepared from the same prepared MF slices were all under 2%, which revealed high repeatability of the method.

#### 3.2.3. The Methodological Validation of the Novel QAMS Method for Determination of Ten Flavone Glycosides in MF

Vitexin was chosen as the reference compound, *fx* of the other nine flavone glycosides including vicenin-2, isoschaftoside, schaftoside, 2”-O-rahmnosylvitexin, isovitexin, isoviolanthin, nicotiflorin, astragalin, and narcissoside were 1.04, 0.60, 0.76, 0.63, 1.04, 0.80, 0.91, 1.13, and 2.40, respectively (Table 2). Then the contents of them were calculated according to the equation shown above by QAMS. As shown in Table 3, the relative errors between the two methods were all less than 5%, indicating the detective efficiency of QAMS was equal to that of ESM. It confirmed that the novel QAMS method could be used for quantitative profiling of these ten components in MF.

### 3.3. Quantitative Flavone Glycosides Profiles Were Different among 21 Batches of MF

**Figure 5:**
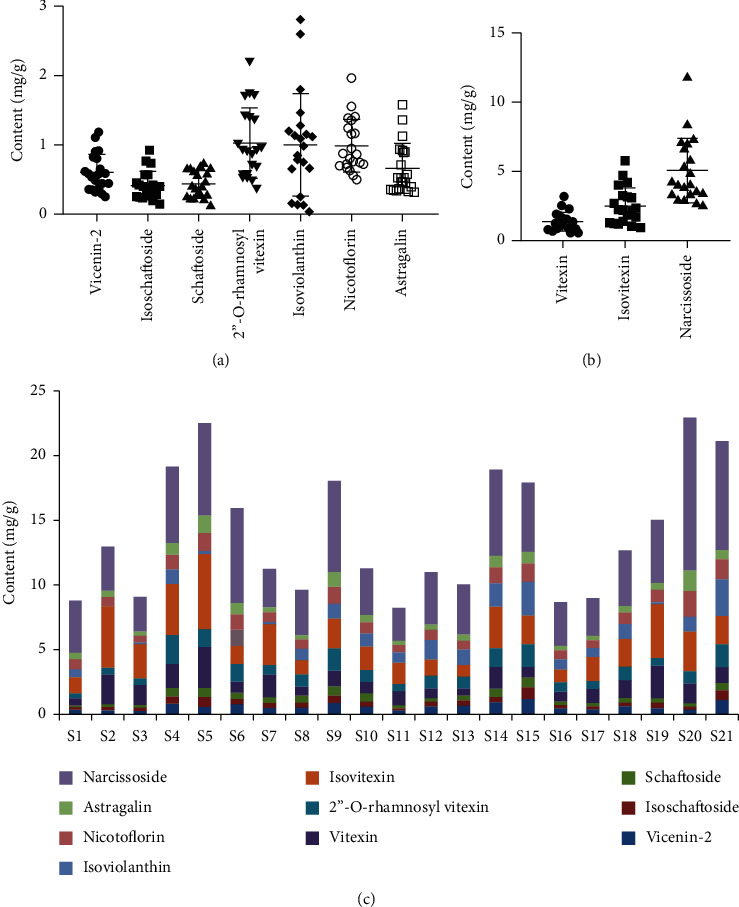
The contents of ten flavone glycosides in the 21 batches of prepared Microctis Folium slices. (a-b) The contents of ten compounds range 0–3 mg/g and 0–15 mg/g. (c) The stacked column of ten flavone glycosides in 21 batches of samples.

It is well known, for Chinese medicine, the prepared slices of crude herbal drugs supplied by the hospital pharmacies can be directly used in clinic. No matter how complicated situation the handling and transportation process is, it is critical that the well-prepared slices of MF must have adequate active ingredients. Therefore, quantitative profiling of the prepared MF slices is regarded as the most efficient for its quality control. In this study, ten glycosides in 21 batched of prepared MF slices collected from different hospital pharmacies were quantified according to the peak area of each compound by QAMS (Figure 5). The results showed that the mean content of narcissoside was the highest at 5.04 mg/g in MF followed by isovitexin (2.49 mg/g) and vitexin (1.35 mg/g) (Figure 5(b)). Meanwhile, the other seven flavonoids made up smaller proportion of that (Figure 5(a)). Moreover, the contents of vicenin-2, isoschaftoside, schaftoside, isoviolanthin, nicotiflorin, astagalin, and narcissoside in the 21 batches of samples were slightly dispersive in the plots; they might be the inducement of the variation between different samples. These results were in accordance with the stacked column (Figure 5(c)). Besides, what counted in this column was that the total contents of ten flavonoids showed obvious differences in different samples. For example, the total contents of ten flavonoids in S5 (22.53 mg/g) and S20 (22.96 mg/g) were much higher compared with the other samples. The bottom four samples were S1, S11, S16, and S17, which counted 8.80, 8.22, 8.68, and 8.99 mg/g, respectively.

**Figure 6:**
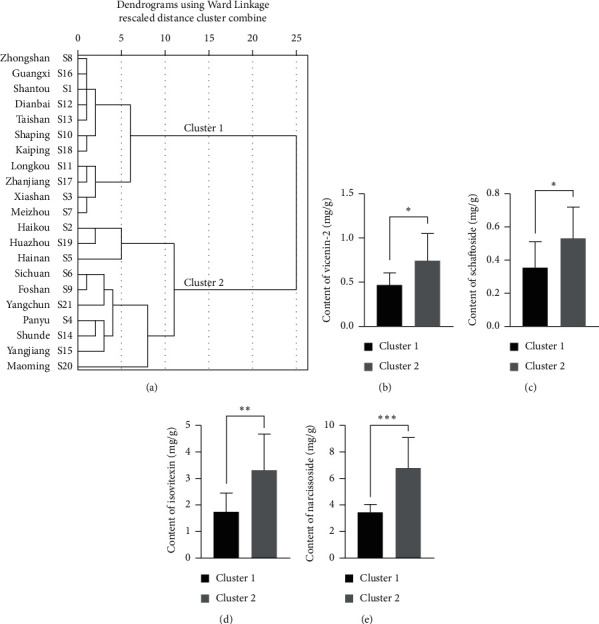
Content variations of the ten flavone glycosides in 21 batches of prepared Microctis Folium slices. (a) Dendrograms of hierarchical cluster analysis by using Ward Link method. (b–d) Contents of vicenin-2, schaftoside, isovitexin, and narcissoside in the two clusters obtained by hierarchical cluster analysis.

In order to show the clear classification of the samples and the relationship among the samples, hierarchical cluster analysis (HCA) was performed [33]. The dendrograms (Figure 6(a)) were constructed according to HCA based on Euclidean distance by using Ward Link method. The HCA results demonstrated significant variations in these ten flavone glycosides among the 21 batches of prepared MF slices collected from different pharmacies. It showed that the 21 batches of samples were divided into two distinct clusters: cluster one consisted of 11 samples and cluster two included 10 samples. As shown in Figures 6(b)–6(e), the contents of vicenin-2, schaftoside, isovitexin, and narcissoside were significantly different between the two clusters. These data confirmed the variations of flavone glycosides profile among different prepared MF slices, which underscored the urgency on its quality control.

All these results indicated that the contents of flavone glycosides caused differences in samples of MF from various clinical pharmacies. The chemical variation may be caused by a lot of factors, such as their genetic origin, growing environment, time to collection, process of production, and storage condition. To assure the efficiency of clinical medicine and the quality of cool herbal tea, quality control of MF was in sore need. Taking the column scatter plot and the dendrograms results into consideration, isoschaftoside, 2”-O-rhamnosylvitexin, isovitexin, nicotiflorin, astragalin, and narcissoside could be the quality markers (Q-markers) to evaluate the quality of MF.

## 4. Conclusions

In conclusion, in the present study, ten flavone glycosides were identified in MF by UPLC-ESI-qTOF-MS/MS and a novel QAMS method was established for their quantification. In addition, quantitative profiling and statistical analysis of 21 batches samples of prepared MF slices revealed the significant variations of the flavone glycosides profiles. Our results hinted that it was necessary to take quality control of MF into account for ensuring its clinical efficacy, and the novel QAMS should be beneficial for the quality control of prepared MF slices and its derived products.

## Figures and Tables

**Table 1:** Ten flavone glycosides identified in Microctis Folium by UPLC-ESI-qTOF-MS/MS.

Peak.no	*t*_R_ (min)	Molecular formula	[M-H]^−^ (Da)	Error (ppm)	HPLC-ESI-MS/MS (m/s) [P-ion^a^ (%)]	Compound
1	10.541	C_27_H_30_O_15_	593.1517	0.843	593^b^: 443 (100), 309 (90), 577 (50), 241 (50)	Vicenin-2
2	11.723	C_26_H_28_O_14_	563.1426	3.552	563^b^: 339 (100), 193 (50), 539 (30), 519 (29)	Isoschaftoside
3	12.330	C_26_H_28_O_14_	563.1413	1.243	563^b^: 289 (100), 385 (55), 539 (33), 367 (30)	Schaftoside
4	13.968	C_21_H_20_O_10_	431.0989	0.232	431^b^: 311 (100), 289 (33), 425 (30), 341 (20)	Vitexin
5	14.123	C_27_H_30_O_14_	577.1575	2.079	577^b^: 431 (100), 311(90), 289 (70), 425 (68)	2”-O-rhamnosyl Vitexin
6	14.278	C_21_H_20_O_10_	431.0991	1.624	431^b^: 311 (100), 341 (40), 289 (18), 407 (8)	Isovitexin
7	14.948	C_27_H_30_O_14_	577.1578	2.664	577^b^: 463 (100), 301 (41), 473 (30), 503 (18)	Isoviolanthin
8	15.825	C_27_H_30_O_15_	593.1514	0.337	593^b^: 463 (100), 289 (65), 387 (30), 581 (60)	Nicotiflorin
9	17.547	C_21_H_20_O_11_	447.0934	0.223	447^b^: 285 (100), 284 (90), 359 (75), 329 (65)	Astragalin
10	17.699	C_28_H_32_O_16_	623.1617	0.160	623^b^: 315 (100), 503 (55), 539 (45), 575 (30)	Narcissoside

^a^P-ion (%), the product ions and the relative intensity of base peak. ^b^Precursor ions. *t*_R_, retention time.

**Table 2:** Linear regression equation and *f*_*x*_ of ten identified flavone glycosides.

Flavone glycosides	*f*_*x*_	Equation	*R*	Range (*μ*g/mL)
Vicenin-2	1.04	*Y* = 2.27 × 10^7^*X* − 4530.71	0.9994	1.25–12.50
Isoschaftoside	0.60	*Y* = 3.83 × 10^7^*X* − 4504.44	0.9999	1.50–15.00
Schaftoside	0.76	*Y* = 3.00 × 10^7^*X* − 3267.77	0.9998	1.31–13.12
Vitexin	1	*Y* = 2.25 × 10^7^*X* + 975.72	0.9998	1.46–14.62
2”-O-rhamnosylvitexin	0.63	*Y* = 9.27 × 10^6^*X* + 5065.10	0.9997	2.24–22.40
Isovitexin	1.04	*Y* = 2.16 × 10^7^*X* − 2002.88	0.9999	1.48–14.76
Isoviolanthin	0.80	*Y* = 1.69 × 10^7^*X* − 10972.54	0.9999	3.2–32.00
Nicotiflorin	0.91	*Y* = 1.64 × 10^7^*X* + 16460.98	0.9999	1.76–17.60
Astragalin	1.13	*Y* = 2.05 × 10^7^*X* − 1813.25	0.9999	1.41–14.12
Narcissoside	2.40	*Y* = 9.37 × 10^6^*X* − 496.55	0.9999	1.61–16.12

**Table 3:** Contents of ten flavone glycosides determined by QAMS and ESM.

Sample no	Vitexin (mg/g)	Vicenin-2			Isoschaftoside			Schaftoside			2”-O-rhamnosylvitexin			Isovitexin			Isoviolanthin			Nicotiflorin			Astragalin			Narcissoside		
		QAMS	ESM	*δ*	QAMS	ESM	Δ	QAMS	ESM	*δ*	QAMS	ESM	*δ*	QAMS	ESM	*δ*	QAMS	ESM	*δ*	QAMS	ESM	*δ*	QAMS	ESM	*δ*	QAMS	ESM	*δ*
S1	0.56	0.36	0.34	0.040	0.19	0.19	0.002	0.14	0.14	0.002	0.36	0.34	0.056	1.26	1.26	−0.001	0.65	0.66	−0.012	0.76	0.76	0.003	0.47	0.47	0.001	4.05	4.06	−0.002
S2	2.29	0.32	0.31	0.040	0.25	0.25	0.006	0.24	0.23	0.010	0.52	0.50	0.047	4.71	4.70	0.002	0.04	0.05	−0.174	0.70	0.70	0.006	0.52	0.52	0.002	3.37	3.37	0.000
S3	1.55	0.25	0.24	0.036	0.25	0.25	0.006	0.23	0.22	0.010	0.48	0.46	0.049	2.67	2.67	0.001	0.13	0.15	−0.091	0.50	0.49	0.018	0.36	0.36	0.000	2.71	2.71	−0.001
S4	1.90	0.82	0.79	0.048	0.57	0.56	0.012	0.62	0.61	0.016	2.20	2.14	0.030	3.99	3.98	0.002	1.09	1.11	−0.015	1.16	1.16	−0.005	0.93	0.93	0.004	5.87	5.87	0.000
S5	3.17	0.58	0.55	0.046	0.77	0.76	0.013	0.70	0.69	0.017	1.39	1.35	0.033	5.79	5.78	0.002	0.25	0.26	−0.054	1.38	1.40	−0.008	1.36	1.35	0.004	7.13	7.13	0.000
S6	0.85	0.80	0.77	0.047	0.40	0.40	0.009	0.48	0.47	0.014	1.36	1.32	0.032	1.37	1.37	0.000	1.28	1.29	−0.008	1.17	1.17	−0.006	0.89	0.89	0.003	7.37	7.38	−0.001
S7	1.82	0.48	0.46	0.044	0.40	0.40	0.010	0.42	0.41	0.014	0.71	0.68	0.041	3.16	3.15	0.001	0.16	0.17	−0.079	0.74	0.74	0.005	0.37	0.37	0.001	2.98	2.98	−0.001
S8	0.66	0.53	0.51	0.044	0.37	0.37	0.009	0.57	0.56	0.015	0.93	0.89	0.036	1.15	1.15	0.000	0.85	0.86	−0.010	0.72	0.72	0.005	0.35	0.35	0.000	3.46	3.46	−0.001
S9	1.23	0.90	0.86	0.048	0.57	0.56	0.011	0.67	0.66	0.016	1.71	1.66	0.031	2.33	2.33	0.001	1.12	1.13	−0.012	1.36	1.37	−0.008	1.13	1.12	0.004	7.03	7.03	−0.001
S10	0.90	0.58	0.55	0.045	0.38	0.38	0.009	0.67	0.66	0.016	0.90	0.87	0.037	1.83	1.83	0.001	0.98	0.99	−0.011	0.88	0.88	0.000	0.57	0.57	0.002	3.61	3.61	−0.001
S11	1.07	0.29	0.28	0.038	0.15	0.15	−0.001	0.29	0.29	0.012	0.52	0.50	0.047	1.69	1.69	0.001	0.78	0.79	−0.016	0.56	0.55	0.013	0.32	0.32	0.000	2.55	2.55	−0.001
S12	0.77	0.59	0.56	0.045	0.38	0.38	0.009	0.29	0.28	0.011	0.95	0.92	0.036	1.29	1.29	0.000	1.46	1.47	−0.006	0.80	0.80	0.002	0.39	0.39	0.000	4.08	4.08	−0.001
S13	0.55	0.65	0.62	0.045	0.42	0.42	0.009	0.41	0.40	0.013	0.86	0.83	0.037	0.94	0.94	−0.001	1.20	1.20	−0.003	0.67	0.67	0.006	0.46	0.46	0.001	3.88	3.89	−0.002
S14	1.67	0.91	0.87	0.048	0.43	0.42	0.010	0.66	0.65	0.016	1.42	1.38	0.033	3.23	3.23	0.001	1.80	1.82	−0.010	1.24	1.25	−0.006	0.90	0.89	0.004	6.67	6.68	0.000
S15	0.86	1.19	1.13	0.049	0.93	0.92	0.013	0.74	0.73	0.016	1.71	1.66	0.031	2.23	2.23	0.001	2.60	2.61	−0.003	1.41	1.42	−0.009	0.91	0.91	0.003	5.37	5.37	−0.001
S16	0.79	0.44	0.42	0.043	0.28	0.28	0.007	0.28	0.27	0.011	0.68	0.65	0.041	1.01	1.02	0.000	0.75	0.76	−0.014	0.72	0.72	0.005	0.34	0.34	0.000	3.39	3.39	−0.001
S17	1.11	0.37	0.36	0.041	0.26	0.26	0.006	0.23	0.23	0.010	0.58	0.55	0.044	1.88	1.88	0.001	0.66	0.68	−0.019	0.63	0.62	0.009	0.32	0.33	0.000	2.94	2.94	−0.001
S18	1.36	0.62	0.59	0.046	0.29	0.29	0.007	0.38	0.38	0.014	1.01	0.98	0.036	2.18	2.18	0.001	1.15	1.17	−0.013	0.87	0.87	0.001	0.52	0.52	0.002	4.30	4.30	−0.001
S19	2.52	0.45	0.43	0.044	0.45	0.45	0.011	0.35	0.34	0.013	0.57	0.54	0.045	4.19	4.18	0.002	0.14	0.15	−0.090	0.95	0.95	−0.001	0.52	0.52	0.002	4.90	4.90	0.000
S20	1.55	0.36	0.35	0.041	0.23	0.23	0.005	0.27	0.26	0.011	0.92	0.89	0.037	3.09	3.08	0.001	1.14	1.15	−0.014	1.97	1.99	−0.012	1.58	1.57	0.004	11.86	11.87	0.000
S21	1.22	1.11	1.05	0.049	0.73	0.72	0.012	0.59	0.58	0.016	1.74	1.69	0.031	2.23	2.23	0.001	2.81	2.82	−0.005	1.56	1.57	−0.010	0.71	0.71	0.003	8.43	8.43	−0.001

## Data Availability

All the raw data will be available from the corresponding author on reasonable request.

## Supplementary Materials

Supplementary Materials Figure S1: 1H-NMR and 13C-NMR spectra of isoschaftoside. 1H-NMR (500 MHz, DMSO-d6) *δ*: 13.67 (1H, s, 5- OH), 8.03 (2H, d, J = 8.8 Hz, H-2', 6'), 6.90 (2H, d, J = 8.8 Hz, H-3', 5'), 6.81 (1H, s, H-3), 4.74 (1H, d, J = 9.9 Hz, H1”'), 4.70 (1H, d, J = 9.6 Hz, H-1”),. 13C-NMR (126 MHz, DMSO-d6) *δ*: 182.85 (C-4), 164.64 (C-2), 161.73 (C-4'), 161.44 (C-5), 158.72 (C-7), 155.61 (C-9), 129.59 (C-2'), 122.08 (C-1'), 116.36 (C-5'), 108.63 (C-6), 105.64 (C-8), 104.27 (C-10), 103.14 (C-3), 82.44 (C-5”'), 79.40 (C-3”'), 74.70 (C-3”), 68.95 (C-2”), 61.73 (C-6”'). Figure S2: 1H-NMR and 13C-NMR spectra of isoviolanthin. 1H-NMR (500 MHz, DMSO-d6) *δ*: 8.04-7.99 (2H, d, H2', 6'), 6.89 (2H, d, J = 8.8 Hz, H-3', 5'), 6.79 (1H, s, H-3), 5.04 (2H, d, J = 1.1 Hz), 5.02-4.97 (8H, m), 4.74-4.72 (4H, m), 4.60 (2H, t, J = 5.7 Hz), 1.29-1.21 (6H, m). 13C-NMR (126 MHz, DMSO-d6) *δ*: 182.21 (C-4), 164.10 (C2), 162.10 (C-4'), 161.22 (C-7), 157.18 (C-5), 155.08 (C-9), 129.03 (C-2', 6'), 121.59 (C-1'), 115.87 (C-3', 5'), 107.10 (C-6), 105.00 (C-8), 103.20 (C-10), 102.39 (C-3), 81.88 (C-5”), 78.74 (C-3”), 77.29 (C-5”'), 74.50 (C-1”'), 74.09 (C-3”'), 73.35 (C-1”), 72.18 (C-2”'), 71.78 (C-4”'), 70.83 (C-2”), 70.68 (C-4”), 61.42 (C-6”), 18.23 (C-6”'). Figure S3: the UPLC-ESI (-)-qTOF-MS total ion current (TIC) of the methanol extract of Microctis Folium. Vicenin-2 (a), isoschaftoside (b), schaftoside (c), vitexin (d), 2”-O-rhamnosylvitexin (e), isovitexin (f), isoviolanthin (g), nicotiflorin (h), astragalin (i), and narcissoside (j). ()
