# Supplementary material for: Variations of Flavone Glycosides Profile Underscore the Necessity of Quality Control of Prepared Microctis Folium Slices
Source: Evid Based Complement Alternat Med. 2020 Nov 26;2020:8887489. doi: 10.1155/2020/8887489 (PMC7718053; doi:10.1155/2020/8887489)
Supplement: Supplementary Materials — Figure S1: 1H-NMR and 13C-NMR spectra of isoschaftoside. 1H-NMR (500 MHz, DMSO-d6) δ: 13.67 (1H, s, 5- OH), 8.03 (2H, d, J = 8.8 Hz, H-2', 6'), 6.90 (2H, d, J = 8.8 Hz, H-3', 5'), 6.81 (1H, s, H-3), 4.74 (1H, d, J = 9.9 Hz, H1”'), 4.70 (1H, d, J = 9.6 Hz, H-1”),. 13C-NMR (126 MHz, DMSO-d6) δ: 182.85 (C-4), 164.64 (C-2), 161.73 (C-4'), 161.44 (C-5), 158.72 (C-7), 155.61 (C-9), 129.59 (C-2'), 122.08 (C-1'), 116.36 (C-5'), 108.63 (C-6), 105.64 (C-8), 104.27 (C-10), 103.14 (C-3), 82.44 (C-5”'), 79.40 (C-3”'), 74.70 (C-3”), 68.95 (C-2”), 61.73 (C-6”'). Figure S2: 1H-NMR and 13C-NMR spectra of isoviolanthin. 1H-NMR (500 MHz, DMSO-d6) δ: 8.04-7.99 (2H, d, H2', 6'), 6.89 (2H, d, J = 8.8 Hz, H-3', 5'), 6.79 (1H, s, H-3), 5.04 (2H, d, J = 1.1 Hz), 5.02-4.97 (8H, m), 4.74-4.72 (4H, m), 4.60 (2H, t, J = 5.7 Hz), 1.29-1.21 (6H, m). 13C-NMR (126 MHz, DMSO-d6) δ: 182.21 (C-4), 164.10 (C2), 162.10 (C-4'), 161.22 (C-7), 157.18 (C-5), 155.08 (C-9), 129.03 (C-2', 6'), 121.59 (C-1'), 115.87 (C-3', 5'), 107.10 (C-6), 105.00 (C-8), 103.20 (C-10), 102.39 (C-3), 81.88 (C-5”), 78.74 (C-3”), 77.29 (C-5”'), 74.50 (C-1”'), 74.09 (C-3”'), 73.35 (C-1”), 72.18 (C-2”'), 71.78 (C-4”'), 70.83 (C-2”), 70.68 (C-4”), 61.42 (C-6”), 18.23 (C-6”'). Figure S3: the UPLC-ESI (-)-qTOF-MS total ion current (TIC) of the methanol extract of Microctis Folium. Vicenin-2 (a), isoschaftoside (b), schaftoside (c), vitexin (d), 2”-O-rhamnosylvitexin (e), isovitexin (f), isoviolanthin (g), nicotiflorin (h), astragalin (i), and narcissoside (j). () [file 8887489.f1.pdf]

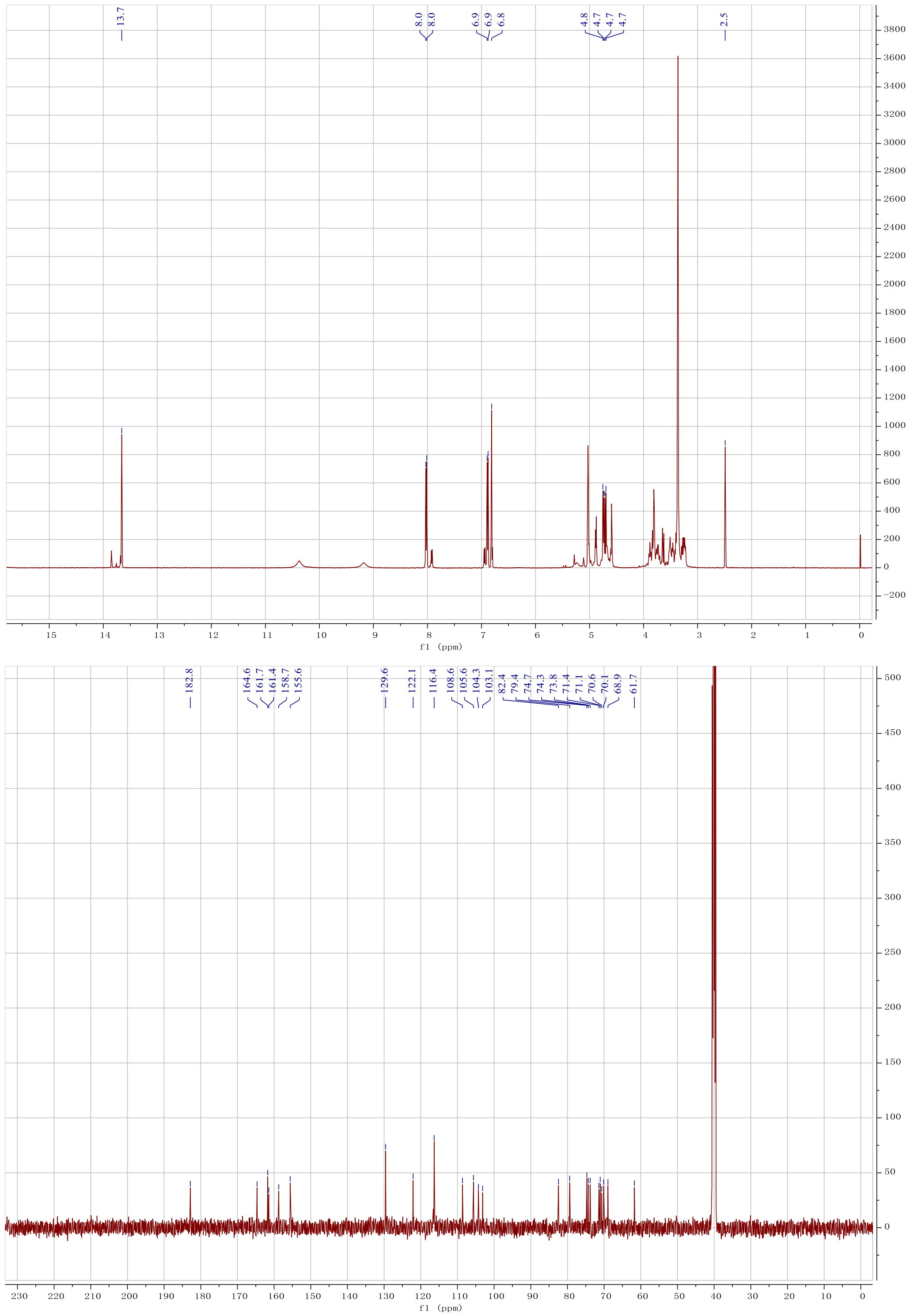

Figure S1. ¹H-NMR and ¹³C-NMR spectra of Isoschaftoside. ¹H-NMR (500 MHz, DMSO-d6) δ: 13.67 (1H, s, 5-OH), 8.03(2H, d, J = 8.8 Hz, H-2',6'), 6.90 (2H, d, J = 8.8Hz, H-3',5'), 6.81 (1H, s, H-3), 4.74 (1H, d, J = 9.9 Hz, H-1'''), 4.70 (1H, d, J = 9.6 Hz, H-1''),. ¹³C-NMR (126 MHz, DMSO-d6) δ: 182.85 (C-4), 164.64 (C-2), 161.73 (C-4'), 161.44 (C-5), 158.72 (C-7), 155.61 (C-9), 129.59 (C-2'), 122.08 (C-1'), 116.36 (C-5'), 108.63 (C-6), 105.64 (C-8), 104.27 (C-10), 103.14 (C-3), 82.44 (C-5'''), 79.40 (C-3'''), 74.70 (C-3''), 68.95 (C-2''), 61.73 (C-6''').

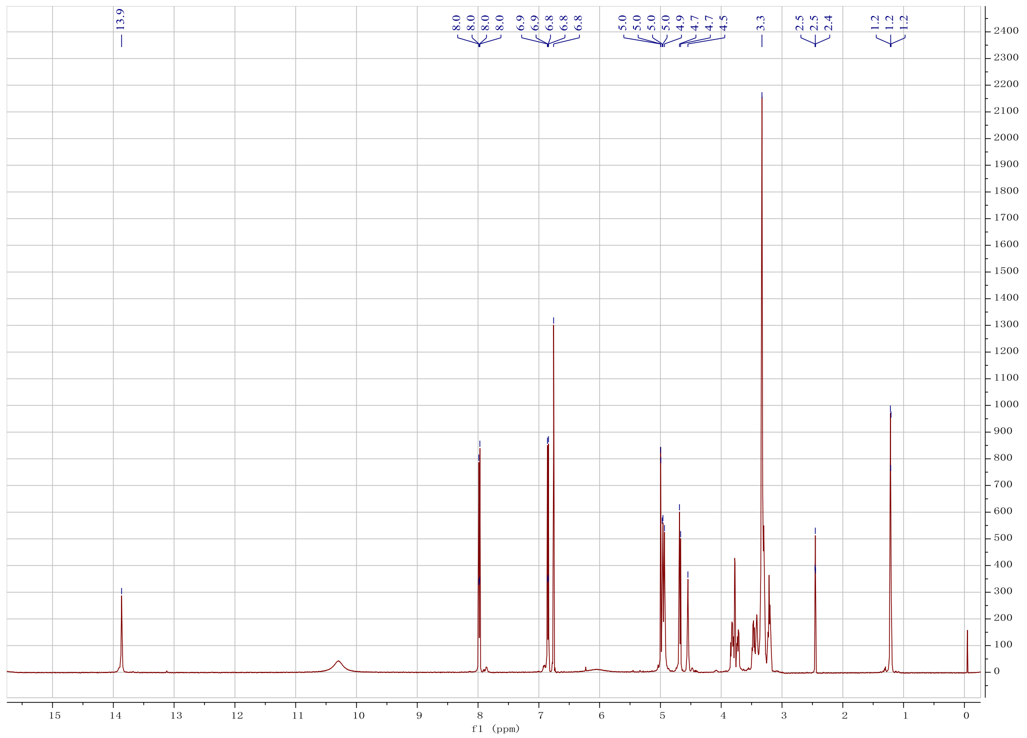

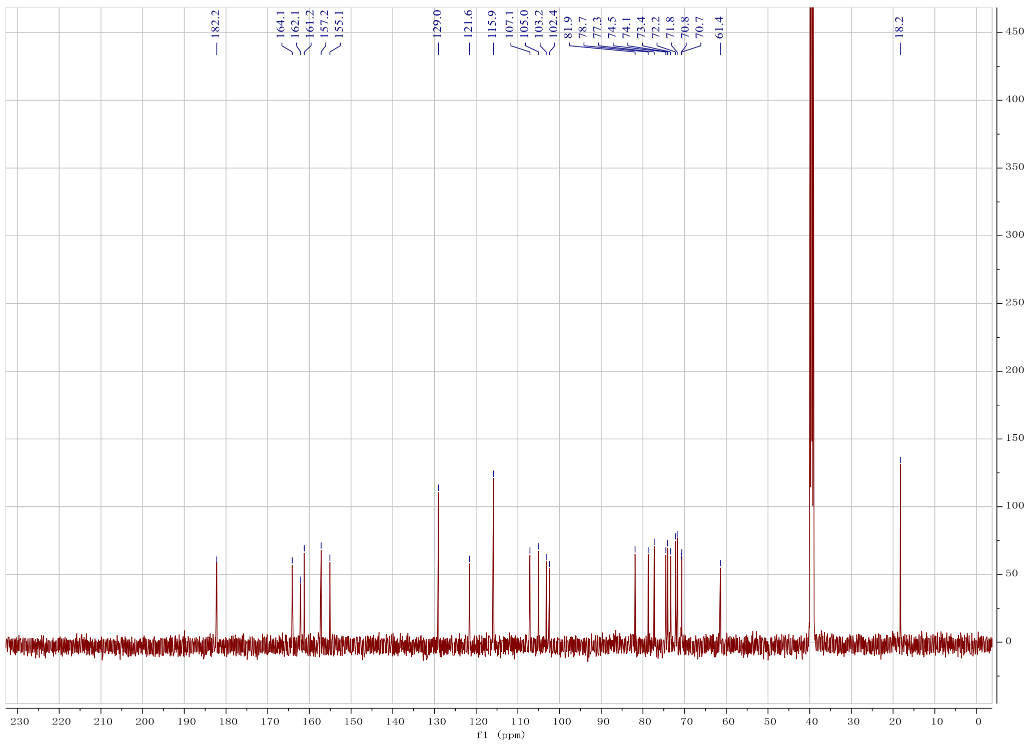

Figure S2 ^{1}H-NMR and ^{13}C-NMR spectra of Isoviolanthin.^{1}H-NMR (500 MHz, DMSO-d6) δ: 8.04-7.99 (2H, d, H-2’, 6’), 6.89 (2H, d, J = 8.8 Hz, H-3’,5’), 6.79 (1H, s, H-3), 5.04 (2H, d, J = 1.1 Hz), 5.02-4.97 (8H, m), 4.74-4.72 (4H, m), 4.60 (2H, t, J = 5.7 Hz), 1.29-1.21 (6H, m). ^{13}C-NMR (126 MHz, DMSO-d6) δ: 182.21 (C-4), 164.10 (C-2), 162.10 (C-4’), 161.22 (C-7), 157.18 (C-5), 155.08 (C-9), 129.03 (C-2’,6’), 121.59 (C-1’), 115.87(C-3’,5’), 107.10 (C-6), 105.00 (C-8), 103.20 (C-10), 102.39 (C-3), 81.88 (C-5”), 78.74 (C-3”), 77.29 (C-5”’), 74.50 (C-1”’), 74.09 (C-3”’),73.35 (C-1”), 72.18 (C-2”’), 71.78 (C-4”’), 70.83 (C-2”), 70.68 (C-4”), 61.42 (C-6”), 18.23 (C-6”’).

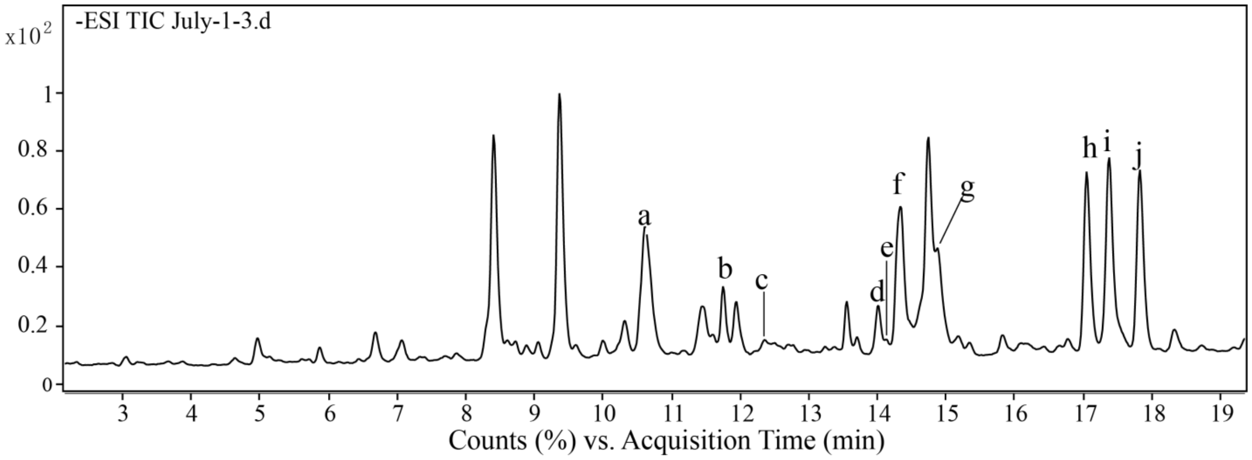

Figure S3. The UPLC-ESI(-)-qTOF-MS total ion current (TIC) of the methanol extract of Microctis Folium. vicenin-2 (a), isoschaftoside (b), schaftoside (c), vitexin (d), 2''-O-rhamnosylvitexin (e), isovitexin (f), isoviolanthin (g), nicotiflorin (h), astragalin (i) and narcissoside (j).
